# Antecedent Avian Immunity Limits Tangential Transmission of West Nile Virus to Humans

**DOI:** 10.1371/journal.pone.0034127

**Published:** 2012-03-23

**Authors:** Jennifer L. Kwan, Susanne Kluh, William K. Reisen

**Affiliations:** 1 Center for Vectorborne Diseases, Department of Pathology, Microbiology and Immunology, School of Veterinary Medicine, University of California Davis, Davis, California, United States of America; 2 Greater Los Angeles County Vector Control District, Santa Fe Springs, California, United States of America; Johns Hopkins University, United States of America

## Abstract

**Background:**

West Nile virus (WNV) is a mosquito-borne flavivirus maintained and amplified among birds and tangentially transmitted to humans and horses which may develop terminal neuroinvasive disease. Outbreaks typically have a three-year pattern of silent introduction, rapid amplification and subsidence, followed by intermittent recrudescence. Our hypothesis that amplification to outbreak levels is contingent upon antecedent seroprevalence within maintenance host populations was tested by tracking WNV transmission in Los Angeles, California from 2003 through 2011.

**Methods:**

Prevalence of antibodies against WNV was monitored weekly in House Finches and House Sparrows. Tangential or spillover transmission was measured by seroconversions in sentinel chickens and by the number of West Nile neuroinvasive disease (WNND) cases reported to the Los Angeles County Department of Public Health.

**Results:**

Elevated seroprevalence in these avian populations was associated with the subsidence of outbreaks and in the antecedent dampening of amplification during succeeding years. Dilution of seroprevalence by recruitment resulted in the progressive loss of herd immunity following the 2004 outbreak, leading to recrudescence during 2008 and 2011. WNV appeared to be a significant cause of death in these avian species, because the survivorship of antibody positive birds significantly exceeded that of antibody negative birds. Cross-correlation analysis showed that seroprevalence was negatively correlated prior to the onset of human cases and then positively correlated, peaking at 4–6 weeks after the onset of tangential transmission. Antecedent seroprevalence during winter (Jan – Mar) was negatively correlated with the number of WNND cases during the succeeding summer (Jul–Sep).

**Conclusions:**

Herd immunity levels within after hatching year avian maintenance host populations <10% during the antecedent late winter and spring period were followed on three occasions by outbreaks of WNND cases during the succeeding summer. Because mosquitoes feed almost exclusively on these avian species, amplification was directly related to the availability of receptive non-immune hosts.

## Introduction

The epidemiology of mosquitoborne arboviral zoonoses is complex. Frequently extensive maintenance and amplification transmission is required prior to spillover or tangential transmission to humans or domestic animals. The efficiency of amplification depends upon the frequency of blood feeding by competent mosquito vectors upon immunologically naïve and competent hosts during favorable climatic conditions [1] that decrease the duration of the gonotrophic cycle increasing the frequency of transmission and that decrease the extrinsic incubation period reducing the chronological age of the vector when transmission can occur [2]. Despite this potential complexity, landscape homogeneity, reduced host and vector diversity, and focused host-selection by the primary vectors frequently simplifies transmission cycles in urban landscapes to a few key species [3]. The population dynamics of these host species, in turn, may dictate the frequency of recurrent outbreaks due to the acquisition and persistence of population or ‘herd’ immunity. Zoonotic mosquito-borne arboviruses seem to rely on two divergent, but often concurrent, strategies for persistence: high virulence/high mortality in amplifying host species that may become regionally depopulated, or moderate virulence/low mortality in host species that acquire herd immunity. Therefore, the timing and intensity of amplification transmission and the occurrence of human outbreaks seems contingent upon host population recruitment to either repopulate or dilute immunity in affected host populations.

The invasion of North America by West Nile virus (family *Flaviviridae*, genus *Flavivirus*, WNV) has provided a unique natural experiment to investigate these processes, because transmission intensity seems greatest in urban/periurban environments where cycles are simplified and frequently involve only a few key vector and avian species [3]. During the invasion of North America, WNV repeatedly has exhibited a three year pattern of silent introduction, explosive amplification to epidemic levels, and then rapid subsidence [4]. Although subsidence may be attributed to multiple factors, immunity within key avian host species seemed critical in slowing or delaying vernal amplification during the year following outbreaks and thereby reducing or preventing spill over or tangential transmission to humans; however, data to substantiate this paradigm has been difficult to obtain. In addition, the levels of herd immunity required for subsidence and recrudescence have yet to be determined. In Los Angeles, California, elevated seroprevalence in key peridomestic maintenance hosts, the House Finch (*Carpodacus mexicanus*) and the House Sparrow (*Passer domesticus*) [5], and concurrent depopulation of the highly susceptible amplifying host, the American Crow [6], [7], were associated with outbreak subsidence during 2005 and low level transmission during subsequent years. Waning seroprevalence in these peridomestic passerines was followed by WNV resurgence to outbreak levels during 2008 and 2011, indicating that there may be thresholds of winter/spring immunity that suppress maintenance transmission, following outbreak years. In agreement, *Culex* bloodmeal identification studies in California repeatedly have documented that during late winter and spring almost all blood meals are taken from House finches and House sparrows [8]–[11]. Before nesting, these populations are composed entirely of after hatching year birds, many of which may have acquired protective immunity during previous seasons.

Late summer communal American Crow roosts may be critical for rapid WNV amplification to outbreak levels, spatially delimiting the distribution of *Culex* infection and human incidence [12], and for seeding virus into residential areas [13], [14], whereas abundant and widely distributed peridomestic passerines may be important as maintenance hosts initiating vernal amplification and continuing epidemic transmission in and around residential habitats. Both House Finches and House Sparrows are competent hosts. Experimentally infected House Finches exhibited viremias >6 log_10_ plaque forming units (PFU)/mL for 4–5 days [15], a titer sufficient to infect *Culex quinquefasciatus*, the main vector present in the Los Angeles area [16]. Mortality in these experimentally infected birds was 65% [15] and field population abundance has been shown to have declined after the arrival of WNV in California [6]. In agreement 26% of dead House Finches submitted for testing to the California Department of Public Health's Dead Bird testing program [17] from Los Angeles were positive for WNV RNA [5]. House Sparrow viremias following experimental infection ranged from 8–10 log_10_ PFU/mL for 4 days in Colorado [18] to 4–6 log_10_ PFU/mL for 2–6 days in California [19], with 38 and 16% mortality, respectively. In agreement, the California Dead Bird program reported that 14% of carcasses from Los Angeles were positive for WNV RNA [5]. Humoral immunity following WNV infection in House Sparrows from Colorado has been demonstrated to last 36 months, with limited decrease in neutralizing antibody titers [20], and similar results were reported for House Finches and House Sparrows from California for up to 8 months [21]. These data indicated that WNV infection should decrease population size and that birds surviving infection should be protected for life from conspecific viral infection thereby dampening subsequent transmission.

Our detailed investigation of WNV epidemiology and ecology in Los Angeles included the systematic monitoring of antibody seroprevalence within House Finch and House Sparrow populations at multiple locations during the 2003–2009 period [5]. Herein, we have extended these data into the 2011 outbreak season, and test the hypothesis that seroprevalence levels in maintenance hosts during late winter determines the efficiency of enzootic amplification of WNV during the subsequent summer season and therefore whether or not an outbreak of human disease will occur. Specifically our study investigated: 1) differences in species specific seroconversion patterns between hatching and after hatching year birds, 2) the impact of WNV infection on the survivorship of banded birds, 3) antibody persistence in naturally infected birds, and 4) the level of herd immunity or seroprevalence necessary to inhibit WNV amplification and human cases. Understanding herd immunity in maintenance host populations is important not only for a better understanding of WNV epidemiology, but also for predicting outbreak risk and organizing preventive intervention in a timely manner.

## Methods

The ecology of the invasion and persistence of WNV in Los Angeles, descriptions of our principal study areas, sampling methods, and temporal and spatial trends in surveillance data from 2003–2008 were summarized previously [5]. The current paper extended our data from 2009 into 2011 and focused on the how the dynamics of WNV infection in House Finch and House Sparrow populations affected tangential transmission to humans.

### Avian Serology

Birds were collected by grain-baited drop-down or Australian crow traps [22], with inlet apertures reduced to limit ingress to small birds. Traps were placed at each of eight sites and were closed for 24 hours biweekly. Birds were aged as juvenile, hatching-year and after hatching-year categories by plumage, and sexed based on plumage [23]. Birds then were banded with USGS bands, and 0.1 ml of blood was collected by 28 g needle syringe from each bird by jugular venipuncture and expelled into 0.9 ml of sterile saline. Samples were clarified by centrifugation and the diluted sera tested by enzyme immunoassay (EIA) for western equine encephalomyelitis virus (WEEV) or flavivirus antibody [24], [25]. Because antibodies against WNV cross-react with closely related St. Louis encephalitis virus (SLEV) [15], EIA results with positive over negative antigen well optical density ratios ≥2 were confirmed and the infecting virus identified by end point plaque reduction neutralization tests (PRNT), using the NY99 strain of WNV and the KERN217 strain of SLEV. Positive PRNTs neutralized >80% of >75 plaque forming units (PFU) of WNV or SLEV grown on Vero cells in 6 well plates at a dilution of ≥1∶20. For specific virus identification, titers exceeded 4× the competing virus.

Serological test results were used to calculate seroprevalence proportions, as the total number of EIA positive birds/total number of birds bled on each bleed date. To estimate seroconversions, new infections were identified as antibody-positive birds known from recapture data to have been previously negative at the most recent previous bleeding. No time period was specified between blood sampling for conversion to an antibody-positive state.

### Sentinel chickens

As described previously [26], flocks of 10 white leghorn hens that were 16–18 weeks of age were deployed annually at each of six sites that were near 6 of the 8 bird sampling sites. Blood samples (0.1 mL) were collected every 2 weeks by brachial venipuncture and placed on filter paper strips [27]. The strips were sent to the California Department of Public Health in Richmond, California, for testing by EIA and immunofluorescence assay (IFA) for presence of antibody to WNV, WEEV, and SLEV [28]. Chickens within flocks were replaced after five or more chickens seroconverted to WNV. Chicken seroconversions previously were found to provide a concordant measure of tangential transmission based on the onset of human cases [26].

### Human case reports

Human cases of West Nile neuroinvasive disease (WNND) were monitored by the Los Angeles County Department of Health and Human Services, Acute Communicable Disease Control, through passive case detection and reporting. WNND cases were limited to those that matched the Center for Disease Control and Prevention (CDC) definition for WNV-associated neuroinvasive illness and had been laboratory-confirmed, typically by demonstration of immunoglobulin M (IgM) antibody in sera or spinal fluid by EIA (http://www.cdc.gov/ncidod/dvbid/westnile/clinicians/clindesc.htm). Febrile cases were not included in our study, because of the progressive decline in testing and reporting after 2004 as indicated by decrease in the ratio of febrile to neuroinvasive cases (data not shown). Additional human infections were discovered through blood donor programs and were included if they developed acute symptoms.

### Analysis

Time series graphs were constructed at monthly intervals for catch per trap-day and seroprevalence. Chi square tests of homogeneity were performed for the birds sampled by infection status and species as well as by site using SAS version 9.2 software (SAS Institute Inc., Cary, NC). To assess the impact of infection history, banded birds were grouped by species and serological status indicating if they were ever infected, and time retained within our study from banding to last recapture. A linear regression was fitted to the numbers collected per 10 week time step transformed by ln (y+1) as a function of time in weeks, presuming constant population loss due to emigration and death. Survivorship was estimated as the backtransformed slope of the fitted regression function.

Time series and correlation analyses of seroprevalence vs. human cases and sentinel chicken seroconversions were used to determine the impact of herd immunity. The herd immunity threshold was defined as the value of seroprevalence that best correlated with the cessation of WNV activity as measured by new WNND cases and sentinel chicken seroconversions. Correlation analyses were performed using SAS version 9.2 Software (SAS Institute Inc., Cary, NC).

### Ethics

The collection, banding, and bleeding of wild birds was done under protocols 11184, 12889 and 15893 approved by the Institutional Animal Care and Use Committee of the University of California, Davis; Master Station Federal Bird Banding permit 22763 issued by the U.S. Geological Survey, California and Resident Scientific Collection permits by the State of California Department of Fish and Game. The husbandry and bleeding of sentinel chickens was done under protocols 11186, 12878 and 15892 approved by the Institutional Animal Care and Use Committee of the University of California, Davis. Use of arboviruses was approved under Biological Use Authorizations #0554 and #0873 issued by the Environmental Health and Safety Committee of the University of California, Davis, and USDA permit #47901. Human data used in this project were granted an exemption from informed consent protocols by the Institutional Review Board at the University of California, Davis (Approval # 201018171-1).

## Results

### Sera collected

**Figure 1:**
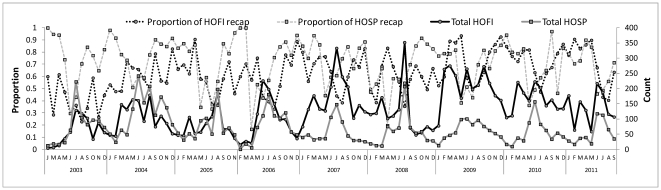
Total numbers of House Finches and House Sparrows collected per month and the proportion banded or recaptured (recap).

**Figure 2:**
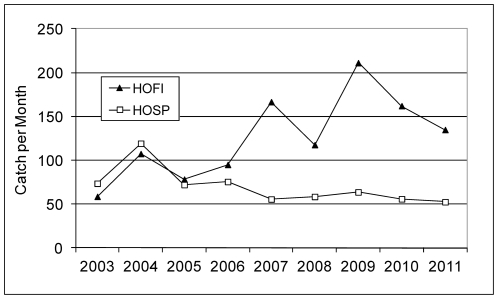
Mean number of House Finches and House Sparrows collected per month during each year.

A total of 22,672 sera were collected from 38 species of birds, of which 87% were House Finches and House Sparrows (Table 1). Other frequently bled birds included small-sized species trapped concurrently, such as Nutmeg Manakins and White-crowned Sparrows, Rock Doves collected as part of bird removal programs, and species such as Mourning Doves sampled at bird rehabilitation centers. House Finches were abundant at all of our sampling locations, whereas most House Sparrows (93%) were collected from 3 of 8 trap locations (Table 2). However, when overall seroprevalence was compared spatially, there were minimal statistical differences. The number collected varied markedly over time (Figure 1), ranging from 13 to 352 House Finches and from 1 to 242 House Sparrows per month, but the catch of these species per month was significantly correlated over time (r = 0.39, df = 95, P<0.01). The number of House sparrows caught per month remained relatively similar among years, whereas there was a progressive increase in the catch of House Finches (Figure 2), leading to a significant species by year interaction term (F = 6.53, df = 8, 184; P<0.001) in a two-way ANOVA comparing species and years. There were no significant temporal relationships among catch per month and the proportions of these birds that were recaptured (Figure 1).

**Table 1:** Number of sera tested (proportion positive for West Nile virus antibodies) in Los Angeles summarized by species and year.

Species	2003	2004	2005	2006	2007	2008	2009	2010	2011	Total
Brown-headed Cowbird	5	70 (0.01)	32 (0.13)	59 (0.05)	77 (0.01)	42	71 (0.03)	64	47 (0.02)	467 (0.03)
California Towhee	6	37 (0.05)	31 (0.03)	49 (0.10)	25 (0.04)	35 (0.06)	23 (0.17)	16	17	239 (0.06)
House Finch	639	1,285 (0.14)	869 (0.26)	1,045 (0.14)	1,943 (0.09)	1,399 (0.14)	2,515 (0.20)	1,766 (0.07)	1,213 (0.03)	12,674 (0.12)
House Sparrow	800	1,416 (0.19)	790 (0.09)	827 (0.04)	670 (0.03)	692 (0.08)	766 (0.08)	615 (0.03)	469 (0.02)	7,045 (0.08)
Mourning Dove	35	86 (0.33)	32 (0.34)	1 (1.00)						154 (0.26)
Nutmeg Manakin	1	6 (0.17)	39 (0.03)	24	90 (0.01)	46 (0.02)	337 (0.01)	322	60	925 (0.01)
Red-winged Blackbird		13 (0.08)	5 (0.20)	2	2					22 (0.09)
Song Sparrow		7	18 (0.11)	3	2	2	1	2		35 (0.06)
White-crowned Sparrow	33	56	58 (0.02)	62	223 (0.01)	131 (0.03)	228 (0.02)	121 (0.02)	89	1001 (0.01)
Totals*	1,524	2,979 (0.16)	1,907 (0.17)	2,100 (0.09)	3,067 (0.07)	2,347 (0.11)	3,945 (0.14)	2,908 (0.05)	1,895 (0.02)	22,672 (0.10)

Only frequently sampled birds included. A more complete listing is presented in Kwan et al. (2010b).

* Included within yearly totals were 8 positives from 110 sera collected from 29 species of birds.

**Table 2:** Total numbers (proportion positive for West Nile virus antibodies) of House Finches and House Sparrows collected at eight study areas in Los Angeles.

Site Name	House Finches	House Sparrows
Machado Lake	1,274 (0.03)	4
Rowland Heights	2,110 (0.13)^A^	5 (0.60)
Whittier Narrows	1,628 (0.12)	2,195 (0.02)
Santa Fe Springs	2,372 (0.25)^B^	2,608 (0.15)
Griffith Park	1,838 (0.12)	170 (0.1)
Sylmar	1,119 (0.01)	29
Santa Clarita	1,494 (0.03)	227
Encino	547 (0.37)^A^^,^ B	1,677 (0.05)

Proportions followed by a letter were significantly different by Chi square test for homogeneity.

LS Means for significant difference.

A p value = 0.04.

B p value = 0.02.

Of the 22,672 sera tested by EIA, 2,267 were positive against flavivirus antigen when tested by EIA, including 1,521 House Finches and 563 House Sparrows (92% of total EIA positives). The proportion of House Finch sera positive for WNV (0.12) was slightly, but significantly (Χ^2^ = 76.4, P<0.0001), greater than the proportion of House Sparrow sera positive (0.08). Mourning doves and other birds from rehabilitation centers frequently were positive during 2004 and 2005, but were sampled inconsistently at low numbers and were not tested after 2005. Other species such as feral Nutmeg Manakins and winter resident White-crowned Sparrows were collected frequently, but rarely were positive (0.01). Of the total EIA positives, 1,946 (87%) were confirmed by PRNT, 112 were PRNT negative, and 209 were not retested. WNV was identified as the infecting virus for all EIA positive birds with PRNT titers ≥1∶40; none had been infected previously with SLEV. The displacement of SLEV by WNV throughout California since 2003 was supported by human case, sentinel chicken serology and mosquito pool diagnostics [5], [29]. Because few other bird species were collected or frequently tested positive, further analyses focused on House Finches and House Sparrows.

### Seroprevalence

**Figure 3:**
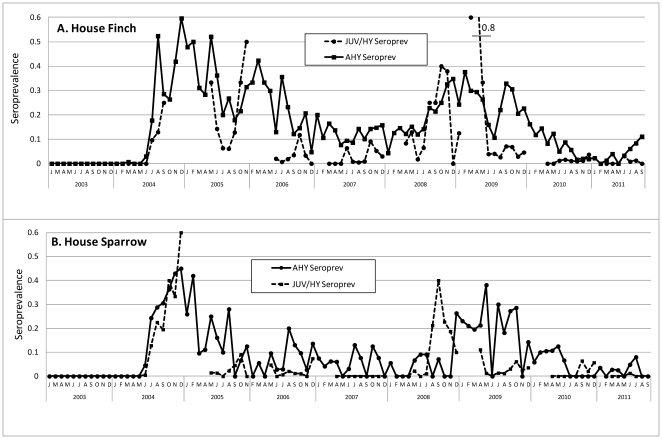
Proportion of after hatching year (AHY) and juvenile/hatching year (JUV/HY) House Finches and House Sparrows positive for antibodies against WNV based on EIA results. Seroprevalence was cumulative and based on all birds regardless of recapture status.

Temporal changes in seroprevalence for young of the year birds classified as juvenile or hatching year and for after hatching year birds are shown in Figure 3 for House Finches and House Sparrows. During the outbreak years of 2004 and 2008 young birds exhibited increased seroprevalence, whereas during intervening years mostly after hatching year birds were seropositive, and the overall seroprevalence levels subsequently declined as these birds were replaced by immunologically naïve hatching year birds. Data shown were seroprevalence by month for different species and age categories, and included birds captured on multiple occasions. We attempted to also show changes in virus activity among years as seroconversions in Table 3. Here, the numbers of banded birds recaptured that previously tested negative were reported by the year that they first tested positive. However, these data were confounded, because the year of first positive recapture was not always well aligned with the year of actual infection, and many AHY House Finches were most likely recorded as seroconversions after their year of infection.

**Table 3:** Total seroconversions/recaptures detected per year for after hatching year (AHY) and juvenile and hatching year (JUV/HY) House Finches and House Sparrows.

House Finches	2004	2005	2006	2007	2008	2009	2010	2011
AHY	48/697	37/415	19/420	26/972	32/654	70/1385	8/1028	8/771
JUV/HY	13/83	6/56	3/134	3/256	10/110	15/446	1/246	1/160

House Sparrows	2004	2005	2006	2007	2008	2009	2010	2011
AHY	37/744	7/336	5/229	3/244	2/134	8/237	1/168	0/185
JUV/HY	22/324	7/181	2/349	0/230	18/295	5/260	1/202	0/99

Seroconversions were listed within the first year they were detected for recaptured birds.

### Survivorship

**Figure 4:**
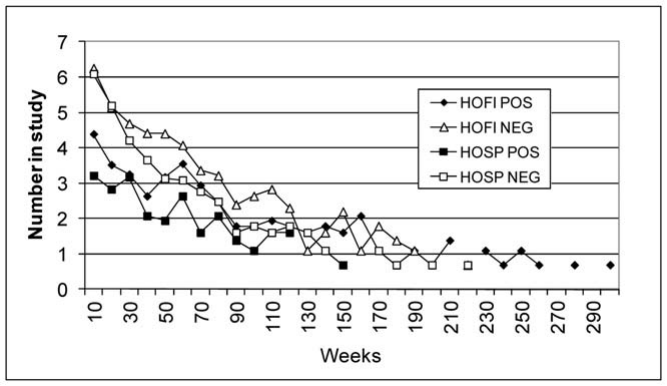
Number of House Finches (HOFI) and House Sparrows (HOSP) ever testing positive (POS) or negative (NEG) for West Nile virus antibodies transformed to ln(y+1) and plotted as a function time retained within our study area grouped into 10 week intervals.

Seroprevalence between outbreak years declined (Figure 3) as a function of recruitment and survivorship. The number of birds recaptured was plotted as a function of weeks between the first and last date of capture grouped by species and infection status and transformed to natural logarithms (Figure 4). Numbers of birds recaptured or surviving per 10 week time interval for each group decreased as a significant linear function (P<0.001) of weeks. Interestingly, the slope values for the fitted regression equations for infected birds of both species were significantly less (P<0.05) than the slope values for non-infected birds, indicating they survived significantly longer due to acquired immune status (Table 4). In a 2-way ANOVA of weeks in study grouped by species and infection status, House finches lived significantly longer (F = 16.65, df = 1, 2383, P<0.001) than House Sparrows, and birds ever positive for WNV infection lived significantly longer (F = 158.5, df = 1, 2383, P<0.001) than never infected birds. In agreement with the similarity in regression slopes, the interaction term in this ANOVA was not significant (P>0.05). Population losses for both infected and non-infected birds included death and emigration; however, the uninfected birds also suffered mortality from their initial WNV infection and may have had a greater emigration rate as HY birds departed the study area after fledging. There were no significant differences in regression slopes between species, so the increase in House Finch abundance (Figure 2) may have been due to enhanced recruitment or the progressive acceptance of our traps as routine feeding stations.

**Table 4:** The numbers of House Finches or House Sparrows that ever tested positive (pos) or negative (neg) for WNV antibody transformed by ln(y+1) and regressed as a function of time retained within study areas grouped in 10 week intervals.

	House Finch		House Sparrow	
Statistic	pos	neg	pos	neg
Intercept	3.480	5.410	3.032	4.709
Slope	−0.011	−0.024	−0.014	−0.023
LL	−0.014	−0.029	−0.013	−0.028
UL	−0.009	−0.020	−0.009	−0.017
R^2^	0.816	0.892	0.805	0.822
Survival	0.989	0.976	0.986	0.978
Mean age	52.6	26.0	43.8	18.3
SE	3.33	0.95	3.68	0.90
n	294	1146	118	825

All slopes were significant (P<0.001) when tested by ANOVA. LL and UL are the lower and upper 95% confidence limits about the slope; slopes with non-overlapping limits were significantly different (P<0.05). R^2^ is the coefficient of determination. Survivorship was estimated as was the backtransformed slope and measured retention within the study, with losses due to mortality and emigration. Mean age was expressed as weeks remaining within the study area.

**Figure 5:**
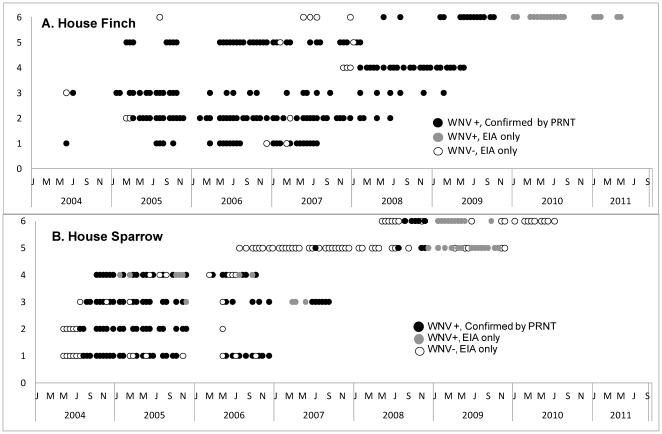
Number of times recaptured birds tested negative, EIA positive, and EIA and PRNT positive. Data are shown for 6 House Finches and 6 House Sparrows collected on multiple occasions.

**Figure 6:**
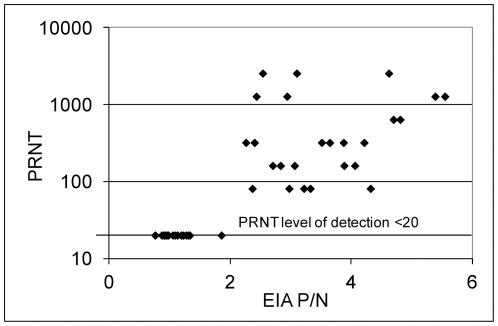
Inverse of plaque reduction neutralization test (PRNT) titers per mL plotted as a function of enzyme immunoassay positive over negative well optical density ratios (EIA P/N) for WNV experimentally infected and uninfected House Finches and House Sparrows (n = 44).

Some long-lived birds were recaptured on multiple occasions over several years (Figure 5). For example, House Finch 2 was captured on 50 occasions and House Sparrow 5 on 57 occasions. These long term recaptures allowed us to examine antibody persistence under field conditions. All 6 of the House Finches that seroconverted remained positive throughout the study, although House Finch 6 lost neutralizing antibody and several birds exhibited unexplained intermittent negative test results. House Sparrows 2, 5 and 6 that were initially positive by both EIA and PRNT reverted to seronegative over time, and all birds exhibited intermittent negative test results. Serum samples were assigned sequential numbers in the field, and laboratory staff did not know the band numbers, so these samples were tested ‘blind’. We initially suspected that these test discrepancies were due to laboratory assay inconsistency; however, when multiple specimens were retested the discrepancies shown in Figure 5 remained. In addition, paired tests from 44 experimentally infected birds that were known to be negative, infected once, or challenged with the same virus provided satisfactory EIA and PRNT results (Figure 6), although none of these birds had infections for longer than 6 weeks.

### Seroprevalence

**Figure 7:**
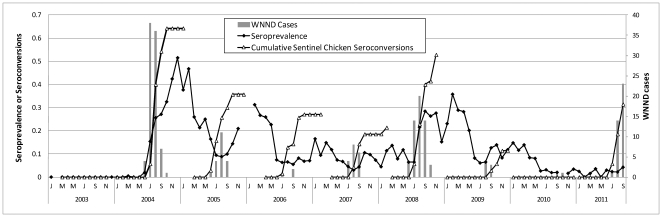
West Nile neuroinvasive disease (WNND) human cases, proportion seroprevalence of House Finches and House Sparrows combined, and cumulative sentinel chicken seroconversions plotted by monthly intervals, Los Angeles, California.

**Figure 8:**
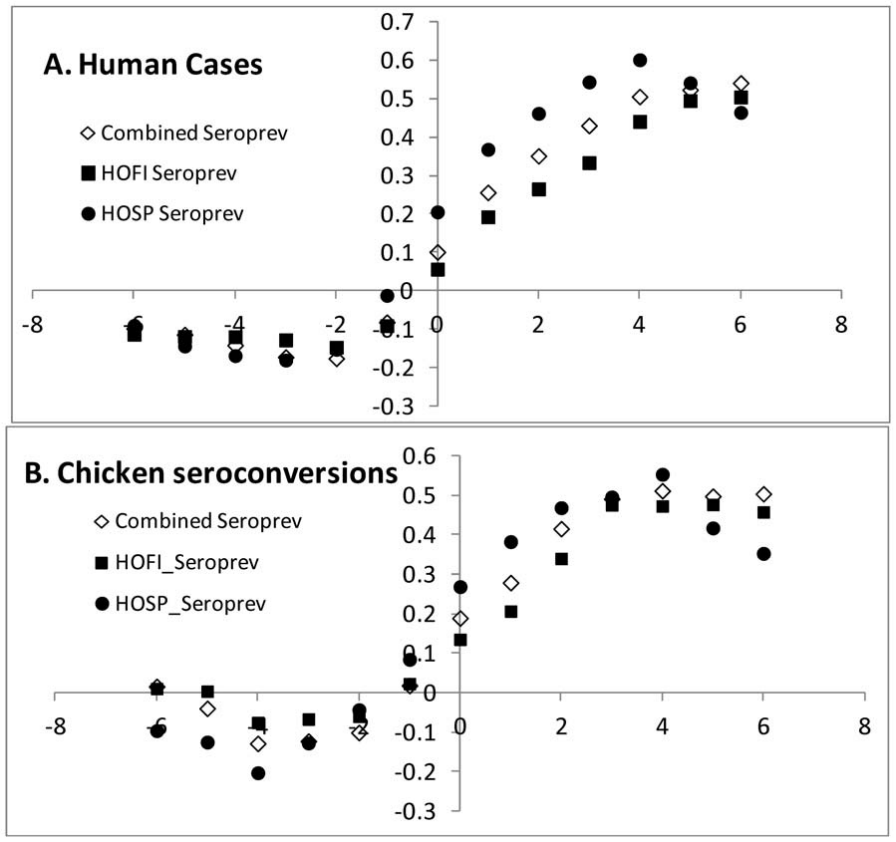
Cross correlations for House Finch (HOFI), House Sparrow (HOSP) and combined seroprevalence against A) Human cases of West Nile neuroinvasive disease and B) sentinel chicken seroconversions.

**Figure 9:**
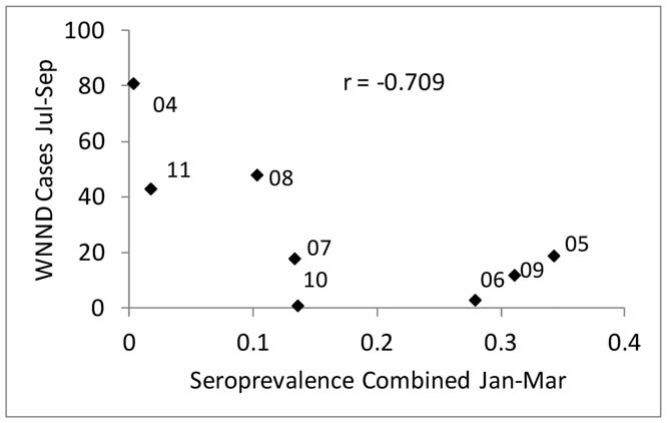
West Nile neuroinvasive disease (WNND) cases during July–September plotted as a function of combined antecedent House Finch and House Sparrow seroprevalence during January–March for each year from 2003–2011. Values were inversely correlated (r, P<0.05).

Results from House Finches and House Sparrows were combined to examine the effects of cumulative seroprevalence or herd immunity on tangential transmission to sentinel chickens and humans (Figure 7). Seroprevalence here was antibody positive birds over total birds bled per month, combined over species and age, and therefore was comparable to the cumulative seroconversions in sentinel chickens within flocks. The increase in seroprevalence commenced concurrent with seroconversions of sentinel chickens and the onset of human cases, but typically peaked 4–6 weeks later, as shown by cross-correlation analyses (Figure 8). It appeared, however, that once seroprevalence or ‘herd immunity’ exceeded ca. 0.25, the numbers of new human cases subsided and remained low during subsequent years until seroprevalence declined to ≤0.10 during late winter/early spring (Figure 9). Overall, the number of WNND cases during the summer transmission season (Jul–Sep) was inversely correlated (r = −0.709, df = 6, P<0.05) with combined seroprevalence during the previous winter (Jan–Mar). Winter seroprevalence ≤0.10 during 2004, 2008 and 2011 was followed by outbreaks of human WNND reported to the Los Angeles Department of Public Health.

## Discussion

Elevated herd immunity in peridomestic House Finch and House Sparrow populations impacted WNV transmission dynamics in Los Angeles in several ways. First, the accumulation of seropositve birds to >25% of the total during outbreak years seemed to dampen or even arrest tangential transmission during late summer (Figure 7), as measured by new WNND cases and seroconversions in sentinel chickens as well as the infection rate in *Cx. p. quinquefasciatus* mosquitoes and in dead American Crows reported by the public [5]. Temperatures in Los Angeles during September and October usually remained warm and conducive to transmission [5], [30], and American Crows at communal roosts remained reasonably abundant, despite mortality due to WNV infection. These data implied that even though viremic corvids may have been critical in driving infection into the *Culex* vector population [12], transmission at large communal roosts may not have been sufficient to continue tangential transmission without a receptive passerine population to support peridomestic transmission [13], [14]. Interestingly, the level of protective herd immunity in these maintenance hosts seen here for a complex zoonotic arbovirus was far less than the estimated 75–85% required for vaccination to protect humans from directly transmitted pathogens [31]. However, further field studies are needed to establish the levels of corvid abundance and infection at late summer communal roosts that are needed to support outbreaks of WNV.

Secondly, although the mechanisms of WNV overwintering in California have not been fully resolved, several paradigms have been supported by field data, including persistent infection in quiescent female and vertically infected *Cx. p. quinquefasciatus* and in chronically infected birds, and continued low level transmission during periods of warm weather [32]. Regardless of the overwintering mechanism, transmission most probably commences in late winter when the weather warms, *Cx. p. quinquefasciatus* resume gonotrophic activity, and resident passerines begin reproductive behavior. At this time most *Culex* in maritime California blood feed on after hatching year (AHY) House Finches and House Sparrows [10], [33], and therefore elevated herd immunity in these species would suppress transmission and delay amplification until after the recruitment of naïve hatching year (HY) birds. As indicated by the reduced number of seroconversions (Table 3) as well as the low seroprevalence in HY birds (Figure 3), years with decreased transmission produced few new infections, and during these subsidence years seroprevalence was associated with surviving AHY birds infected during previous years.

Acquired immunity significantly increased avian survivorship and the mean duration of life within our study areas (Table 4), and may have slowed the decline of seroprevalence following outbreak years, requiring more than one season to dilute seroprevalence to low enough levels to allow early season amplification. In agreement, WNV recrudescence occurred in 2008, 3 years after the 2004 outbreak, and in 2011, 2 years after the 2008 outbreak. The shorter period of subsidence after 2008 may have related to the lower peak seroprevalence during the outbreak (<30%) and the more rapid return to <10% than after 2004, when seroprevalence peaked at 51% during December. Although difficult to measure, both species populations also were probably impacted heavily by mortality associated with WNV infection, because experimentally infected House Finches and House Sparrows showed 65 and 38% mortality, respectively. This mortality may have contributed to the survivorship differences seen between seropositive and negative birds. Interestingly, although calculated differently, our survivorship estimates were greater than those for a smaller cohort of House Finches and House Sparrows banded and recaptured in Kern County [34] when they were infected at a low level with WEEV and SLEV [35]. Similar to our data, they found that House Finches lived longer than House Sparrows, and that some especially long-lived birds were recaptured 55 and 66 months after banding, respectively. In addition, House Finches in Sacramento County were found to have an annual survival rate of 0.59 before and 0.47 after the arrival of WNV [36]. Annual survivorship estimates for seropositive birds in LA were similar to pre-WNV estimates in Sacramento of 0.59, but estimates for Los Angeles seronegative birds (0.35) were much less than post-WNV estimates of 0.47 per year in Sacramento, perhaps reflecting the impact of greater infection rates in Los Angeles.

Antibody persistence waned over time in naturally infected birds, contrasting laboratory studies [20], [37] and outdoor flight cage studies [21] that showed long term retention of PRNT titers in House Sparrows and House Finches. Field data for 12 especially long-lived birds showed that some individuals intermittently reverted to antibody-negative over time, agreeing with previous results for SLEV in naturally-infected field birds [38]. Our short term field data for House Finches and House Sparrows agreed well with several laboratory host competence experiments [15], [19] that showed good agreement between EIA and PRNT results for up to 6 weeks. Although data coding errors by mis-reading band numbers in the field cannot be discounted or double checked, it appears that some birds may undergo changes in immunity with age leading to changes in test results. Future studies will address the impact of these immune changes on virus recrudescence in chronically infected birds.

In addition to ambient temperature [2], the level of herd immunity within peridomestic passerine populations during late winter and spring seemed critical in delineating the timing and slope of the WNV amplification curve, in establishing the amplitude of the curve during summer, and ultimately in determining if sufficient tangential transmission occurred to precipitate an outbreak of human disease. Although these conclusions were well-supported by data for Los Angeles, additional studies are needed in other habitats such Bakersfield in Kern County where outbreaks have recurred during successive years despite moderate herd immunity in House Finches and Western Scrub-jays [39] or in habitats with high avian diversity and low corvid abundance such as Coachella Valley [29] where continued low herd immunity has failed to result in outbreaks of human disease.
